# The potential of anti-malarial compounds derived from African medicinal plants, part III: an *in silico* evaluation of drug metabolism and pharmacokinetics profiling

**DOI:** 10.1186/s13588-014-0006-x

**Published:** 2014-09-05

**Authors:** Pascal Amoa Onguéné, Fidele Ntie-Kang, James Ajeck Mbah, Lydia Likowo Lifongo, Jean Claude Ndom, Wolfgang Sippl, Luc Meva′a Mbaze

**Affiliations:** 1 Department of Chemistry, Faculty of Science, University of Douala, Douala, 00237 Cameroon; 2 Department of Chemistry, Faculty of Science, Chemical and Bioactivity Information Centre, University of Buea, Buea, 00237 Cameroon; 3 Department of Pharmaceutical Sciences, Martin-Luther University of Halle-Wittenberg, Wolfgang-Langenbeck Str. 4, Halle (Saale), 06120 Germany

**Keywords:** Africa, Malaria, Medicinal plants, Metabolism, Natural products, Pharmacokinetics

## Abstract

**Background:**

Malaria is an endemic disease affecting many countries in Tropical regions. In the search for compound hits for the design and/or development of new drugs against the disease, many research teams have resorted to African medicinal plants in order to identify lead compounds. Three-dimensional molecular models were generated for anti-malarial compounds of African origin (from 'weakly' active to 'highly' active), which were identified from literature sources. Selected computed molecular descriptors related to absorption, distribution, metabolism, excretion and toxicity (ADMET) of the phytochemicals have been analysed and compared with those of known drugs in order to access the 'drug-likeness' of these compounds.

**Results:**

In the present study, more than 500 anti-malarial compounds identified from 131 distinct medicinal plant species belonging to 44 plant families from the African flora have been considered. On the basis of Lipinski's 'Rule of Five', about 70% of the compounds were predicted to be orally bioavailable, while on the basis of Jorgensen's 'Rule of Three', a corresponding >80% were compliant. An overall drug-likeness parameter indicated that approximately 55% of the compounds could be potential leads for the development of drugs.

**Conclusions:**

From the above analyses, it could be estimated that >50% of the compounds exhibiting anti-plasmodial/anti-malarial activities, derived from the African flora, could be starting points for drug discovery against malaria. The 3D models of the compounds have been included as an accompanying file and could be employed in virtual screening.

**Electronic supplementary material:**

The online version of this article (doi:10.1186/s13588-014-0006-x) contains supplementary material, which is available to authorized users.

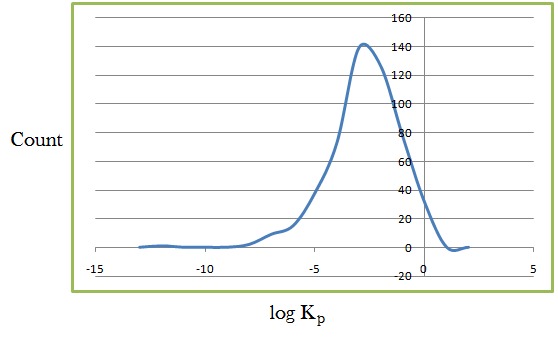

## Background

Malaria is an endemic disease which affects vast proportions of the populations of most Tropical countries (covering Africa, Asia and Latin America) [1],[2]. The disease condition is caused by protozoans of the *Plasmodium* genus, mostly *Plasmodium falciparum*[3]. Statistics show that about half of the world's population is at risk of contracting malaria and that 1 to 2 million annual deaths (mostly amongst African children) can be attributed to malaria alone [2]-[4]. In addition, the spread of the disease has been enhanced by the development of resistance in the anopheline vector against standard insecticides, amongst other factors, which have not unfortunately been put under check [5].

One promising way to fight malaria is to search for vaccines and new drugs, since no vaccine has yet been put in the market and the disease-causing parasites have developed resistant strains against existing chemotherapies [5]-[7]. It should however be mentioned that the process of discovering a drug is quite timely and costly [8]. One of the current approaches for shortening the time required and cutting down the cost for the discovery of lead compounds which potentially inhibit or modulate known drug targets is to incorporate computer-based methods like docking techniques, pharmacophore-based searches and neural networking [9]-[13]. Computer-based methods have also been incorporated in the prediction of likely metabolic pathways of drug molecules, as well as predict their pharmacokinetic profiles [14]-[17]. The absorption, distribution, metabolism, excretion and toxicity (ADMET) profile of a potential drug molecule should be known if it has to stand the chances of entering the market. Hence, assessing such information for lead compounds early enough would help eliminate molecules with predicted uninteresting profiles and eventually cut down the price of drug discovery [8].

With the accumulation of 'wet lab' biodata on drug metabolism and pharmacokinetics (DMPK) by the close of the 1990s, pharmaceutical companies are increasingly switching over to the use of statistical and knowledge-based methods, implemented in computer software, in the prediction of ADMET/DMPK properties of drug leads, in contrast to the former approach which is more costly and time consuming [14]-[17]. In our quest to assess the potential of natural products (NPs) derived from African medicinal plants for the development of anti-malarial drugs [18],[19], an *in silico* approach based on computed molecular descriptors has been carried out, in comparison with those of known drugs, as previously described in the literature [20]-[24]. In this paper, we present a computer-based DMPK analysis of >500 anti-malarial compounds, which have been previously isolated from the African flora.

## Methods

### Data sources

The plant sources, geographical collection sites, chemical structures of pure compounds as well as their spectroscopic data were retrieved from literature sources comprising of MSc theses, PhD theses, textbook chapters and journal articles, with references ranging from 1971 to 2013. A full list of journals consulted is given in the supplementary material (Additional file 1). By convention, activities were categorized into 'very potent', 'good', 'good to moderate', 'weak', 'very weak' and 'inactive'. Following the criteria used by Mahmoudi et al. [25] and Wilcox et al. [26], a pure compound was considered highly active if IC_50_ < 0.06 μM, being active with 0.06 μM ≤ IC_50_ ≤ 5 μM, weakly active when 5 μM ≤ IC_50_ ≤ 10 μM, and compounds with IC50 > 10 μM were considered inactive. The following inhibition percentages were proposed for *in vivo* activity of anti-malarial extracts at a fixed dose of 250 mg kg^−1^ day^−1^: 100% to 90% (very good activity), 90% to 50% (good to moderate), 50% to 10% (moderate to weak) and 0% (inactive) [27].

### Generation of 3D models, optimization and correction of protonation states

The 2D structures of the compounds were retrieved from the literature sources, and all 3D molecular models were generated using the graphical user interface (GUI) of the MOE software [28] running on a Linux workstation with a 3.5 GHz Intel Core2 Duo processor. The 3D structures were generated using the builder module of MOE, and energy minimization was subsequently carried out using the MMFF94 force field [29] until a gradient of 0.01 kcal mol^−1^ was reached. The 3D structures of the compounds were then saved as .mol2 files subsequently treated with LigPrep [30]. This implementation was carried out with the GUI of the Maestro software package [31], using the optimized potentials for liquid simulations (OPLS) forcefield [32]-[34]. Protonation states at biologically relevant pH were correctly assigned (group I metals in simple salts were disconnected, strong acids were deprotonated, strong bases protonated, while topological duplicates and explicit hydrogens were added). The generated 3D models have been included in the supplementary material (Additional file 2).

### Calculation of molecular descriptors

A set of ADMET-related properties (a total of 46 molecular descriptors) were calculated by using the QikProp program [35] running in normal mode. QikProp generates physically relevant descriptors and uses them to perform ADMET predictions. An overall ADME-compliance score - drug-likeness parameter (indicated by #stars) - was used to assess the pharmacokinetic profiles of the compounds. The #stars parameter indicates the number of property descriptors computed by QikProp that fall outside the optimum range of values for 95% of known drugs. The methods implemented were developed by Jorgensen and Duffy [36]-[38]. Some of the computed ADMET descriptors are shown in Additional file 3: Table S1, along with their significance in DMPK profiling and the recommended ranges for 95% of known drugs.

## Results and discussion

### Plant families and compound types

**Figure 1:**
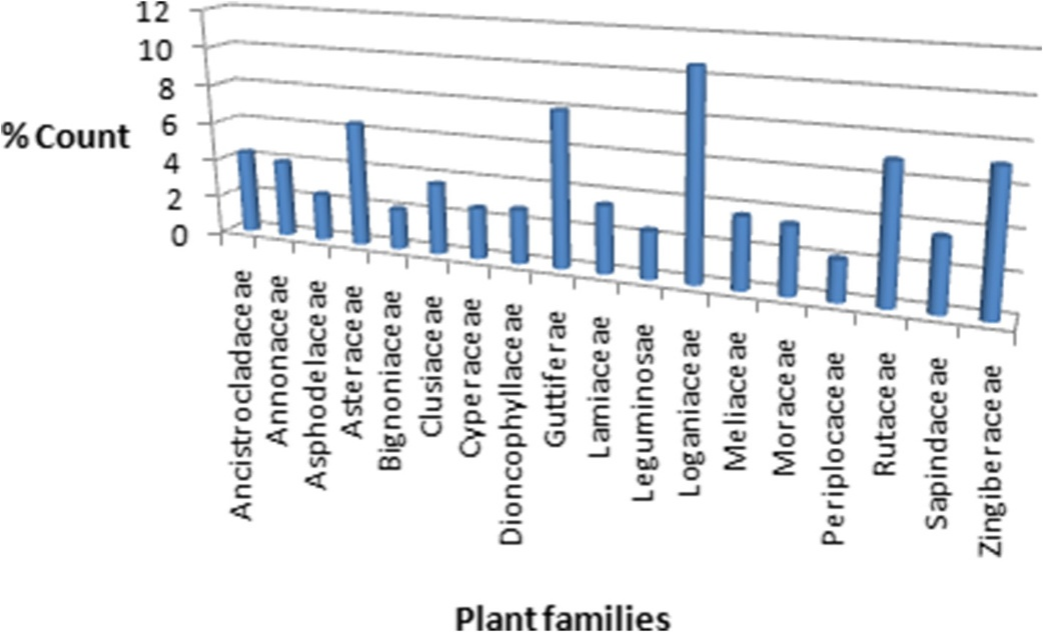
**Bar chart showing the distribution of the 511 anti-malarial compounds by plant family of origin.**

**Figure 2:**
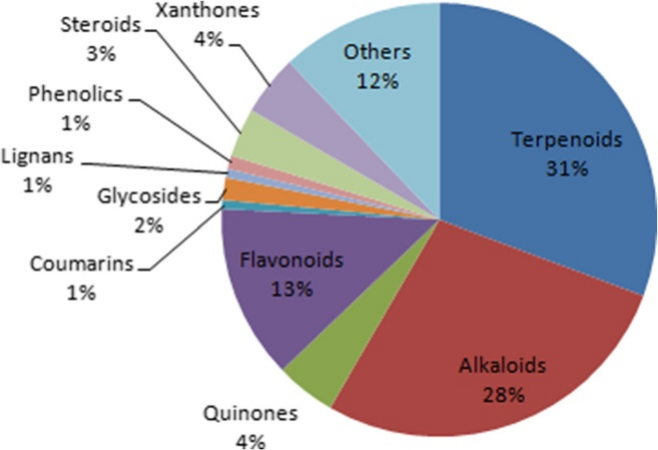
**Pie chart showing the classification by compound types.**

From this survey, 511 anti-malarial compounds were identified from 131 plant species belonging to 45 families, a majority being isolated from the Loganiaceae family (10.6%). Significant proportions were also identified from the Guttiferae, Zingiberaceae, Rutaceae, Asteraceae, Ancistrocladaceae, Annonaceae, Clusiaceae, Meliaceae, Sapindaceae, Lamiaceae, Moraceae, Dioncophyllaceae, Cyperaceae, Asphodelaceae, Leguminosae, Periplocaceae and Bignoniaceae families, with respective percentage compound counts of 8.0%, 7.1%, 6.9%, 6.4%, 4.3%, 4.0%, 3.6%, 3.6%, 3.6%, 3.5%, 3.5%, 2.8%, 2.6%, 2.4%, 2.4%, 2.1% and 2.1% of identified anti-malarials (Figure 1). Less than 2% of the anti-malarials were isolated from each of the remaining plant families. A majority of the compounds were terpenoids (30.7%), followed by alkaloids, flavonoids, quinones and xanthones, respectively representing 27.7%, 12.9%, 4.5% and 4.5% by compound count (Figure 2). Only 20 compounds were identified with *in vivo* anti-malarial activities, while 278 compounds showed *in vitro* activities from moderate to very high activities. These have been discussed in our previous review articles in this series [18],[19].

### Significance of selected computed molecular descriptors

Selected molecular descriptors, along with the recommended range of values for 95% of known drugs, as computed by the QikProp program [35], have been shown in Additional file 3: Table S1. According to Lipinski's 'Rule of Five', a compound is likely to be orally available when the molecular weight (MW) <500, the logarithm of the octan-1-ol/water partition coefficient (log P) < 5, the number of hydrogen donors (HBD) ≤5 and the number of hydrogen bond acceptors (HBA) ≤10 [39]. Compounds which comply with this rule are considered to be drug-like. Moreover, Jorgensen's 'Rule of Three' has often been used as a criterion to predict oral availability of drugs [40]. According to this rule, a compound will more likely be orally available when all or some of the rules, the logarithm of the predicted aqueous solubility (log *S*_wat_) > −5.7, the predicted Caco-2 permeability (BIP_Caco − 2_) >22 nm s^−1^ and the number of primary metabolites <7, are respected. The solubility calculation procedure implemented depends on the similarity property space between the given molecule and its most similar analogue within the experimental training set used to develop the model implemented in QikProp; i.e., if the similarity is <0.9, then the QikProp predicted value is taken, otherwise, the predicted property, *P*_pred_, is adjusted such that

1 $$P_{\mathrm{pred}}=SP_{\text{exp}}+\left(1-S\right)P_{\mathrm{QP}}$$

where *S* is the similarity, and *P*_exp_ and *P*_QP_ are the respective experimental and QikProp predictions for the most similar molecule within the training set. In Equation 1, if *S* = 1, then the predicted property is equal to the measured experimental property of the training set compound. The predicted apparent Caco-2 cell permeability, BIP_Caco − 2_ (in nm s^−1^), models the permeability of the gut-blood barrier (for non-active transport), even though this parameter is not often correctly predicted computationally [41]. Moreover, a molecule's size, its capacity to make hydrogen bonds, its overall lipophilicity, and its shape and flexibility are important properties to consider when determining permeability. Molecular flexibility has been seen as a parameter which is dependent on the number of rotatable bonds (NRB), a property which influences bioavailability in rats [42].

The overall drug-likeness of a molecule is often determined by the #stars parameter, which depends on 24 computed parameters (descriptors) of a molecule with respect to the recommended range for 95% of known drugs (Additional file 3: Table S1). A #star = 0 corresponds to an ideally drug-like molecule, while #stars = *n* indicates that a given molecule has *n* non-compliant descriptors (values fall outside the recommended range for 95% of known drugs). The solubility of a drug, evaluated by the model of Jorgensen and Duffy [36],[37], determines the bioavailability of a drug to an extent, since the bioavailability of a compound depends on the processes of absorption and liver first-pass metabolism [43]. Absorption in turn depends on the solubility and permeability of the compound, as well as interactions with transporters and metabolizing enzymes in the gut wall.

The computed blood/brain partition coefficients (log *B*/*B*) of drug molecules are often used as indicators to predict access to the central nervous system (CNS). This is because too polar drugs do not cross the blood/brain barrier (BBB). In addition, Madin-Darby canine kidney (MDCK) monolayers are widely used to make oral absorption estimates, the reason being that these cells also express transporter proteins but only express very low levels of metabolizing enzymes [42]. They are also used as an additional criterion to predict BBB penetration. Thus, our calculated apparent MDCK cell permeability could be considered to be a good mimic for the BBB (for non-active transport). The predicted skin permeability parameter (log *K*_p_) is another important parameter for drug distribution [44],[45]. The predicted maximum transdermal transport rates, *J*_m_ (in μ cm^−2^ h^−1^), were computed from the aqueous solubility (S_wat_) and skin permeability (*K*_p_), using the relation (2), also expresses the efficiency of drug distribution:

2 $$J_{m}=K_{p}\times \mathrm{MW}\times S_{\mathrm{wat}}$$

The efficiency and distribution of a drug may be affected by the degree to which it binds to the proteins within blood plasma. When a drug binds to plasma proteins (like human serum albumin, lipoprotein, glycoprotein, α, β, and γ globulins), the quantity of the drug in general blood circulation is greatly reduced and hence the less bound a drug is, the more efficiently it can traverse cell membranes. The predicted plasma-protein binding has been estimated by the prediction of binding to human serum albumin; the log *K*_HSA_ parameter (recommended range is −1.5 to 1.5 for 95% of known drugs). The number of metabolic steps (#metab) also determines whether the molecules can easily gain access to the target site after entering the blood stream.

The toxicity parameter is often predicted by the logarithm of IC_50_ values for blockage of the human ether-a-go-go related gene (HERG). HERG encodes a potassium ion (K^+^) channel that is implicated in the fatal arrhythmia known as *torsade de pointes* or the long QT syndrome [46]. The HERG K^+^ channel is best known for its contribution to the electrical activity of the heart that coordinates the heart's beating. It therefore appears to be the molecular target responsible for the cardiac toxicity of a wide range of therapeutic drugs [47]. HERG has also been associated with modulating the functions of some cells of the nervous system and with establishing and maintaining cancer-like features in leukemic cells [48]. Thus, HERG K^+^ channel blockers are potentially toxic and the predicted IC_50_ values often provide reasonable predictions for cardiac toxicity of drugs in the early stages of drug discovery [49].

### Lipinski's criteria for evaluation of oral bioavailability

**Figure 3:**
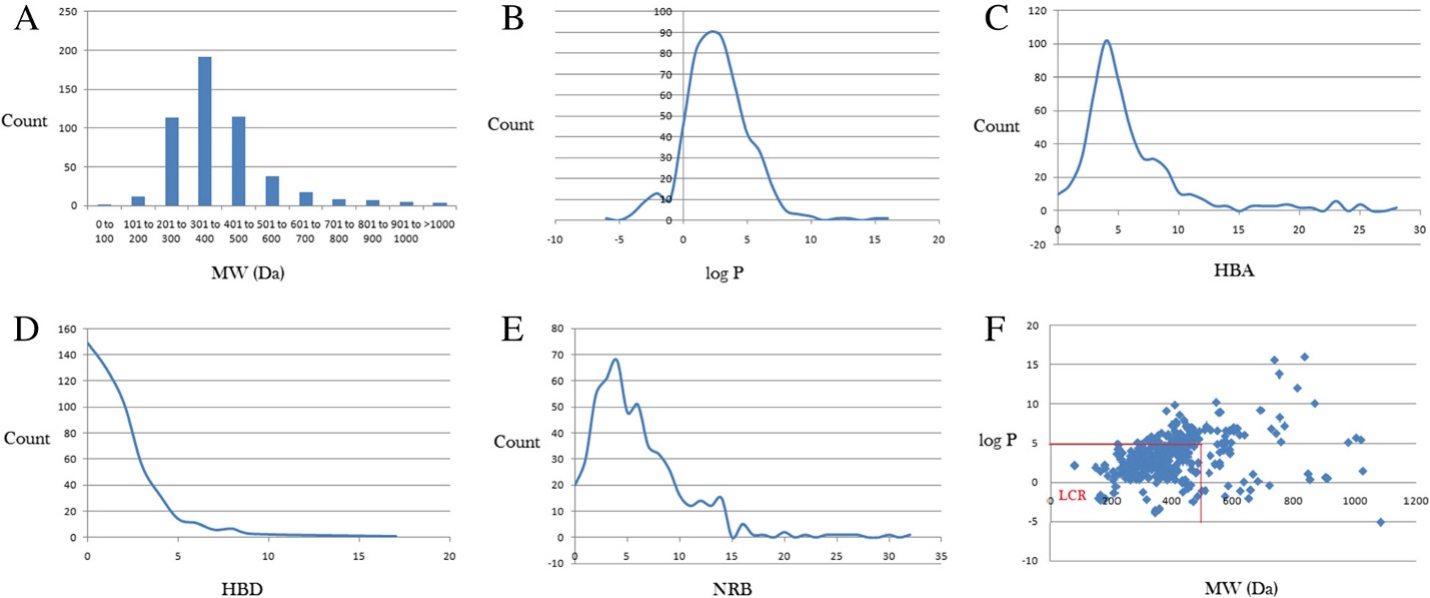
**Graph distribution of Lipinski determinant of 'drug-likeness'. (A)** Bar chart showing distribution of MW. **(B, C, D and E)** Distribution curves of the log P, HBA, HBD and NRB respectively for the 511 anti-malarial compounds from the African flora. **(F)** Scatter plot of MW against log P. For (B), the *x*-axis label is the lower limit of binned data, e.g., 0 is equivalent to 0.00 to 0.99.

Lipinski's 'rule' was extracted from chemical libraries from the World Dug Index (WDI) as a criterion to evaluate likely oral bioavailability [39],[50]. This rule did not take NPs into consideration initially, since Lipinski had postulated that the Rule of Five was not respected by NPs. However, NP libraries have been previously analysed comparatively using this rule in order to have a rough idea of the extent of 'drug-likeness' of a compound library to be used in virtual screening [20]-[22],[51],[52]. In summary, 352 of the 511 compounds analysed showed no violations of Lipinski rules, while 435 compounds showed <2 violations. The maximum number of violations was 3 and the average number was 0.5 (Table 1). This implies that the anti-malarial compounds from the African flora could generally be categorized as 'drug-like' up to approximately 70%. Considering the individual parameters, it was observed that for the MW, 432 out of the 511 compounds analysed complied with Lipinski's criterion (MW < 500 Da), constituting 84.5%. Meanwhile, the greatest proportion of the molecules had MW in the interval 301 ≤ MW ≤ 400, Figure 3A. The log P curve showed a Gaussian-shape, centred on a peak value of 1.5 log P units, with approximately 88% of the compounds complying to Lipinski's criterion (log P < 5), Figure 3B. The graphs of the HBA, HBD and NRB have been shown in Figure 3C,D,E. The number of HBA rose rapidly to a peak value of 4 acceptors, comprising 102 compounds, then fell off rapidly to 28 acceptors for the most complex NPs in this study. The curve for the HBD had a maximum at 0 donors (for 149 of the compounds) and fell off to 1 compound having as many as 17 donors. The distribution for the NRB gave a rugged curve with a peak value at 4 rotatable bonds (RBs), comprising 68 compounds and as many as 32 RBs for the most flexible compound. A scatter plot of MW against log P showed that the region of maximum density of points lay in the interval MW ≤ 500 ∩ log P ≤ 5, defined as Lipinski's compliance region (LCR). A similar trend was seen in the pairwise scatter plots of the other parameters (results not shown).

**Table 1:** **Minimum, maximum and mean values for computed Lipinski parameters**

Lipinski parameter	Minimum	Mean	Maximum
MW (Da)	78.1	394.1	1084.7
log P	−5.1	3.2	16
HBA	0	6.4	28
HBD	0	1.8	17
NRB	0	5.9	32
LV	0	0.5	3

LV, Lipinski violations.

### Jorgensen's criteria for assessment of oral bioavailability

**Figure 4:**
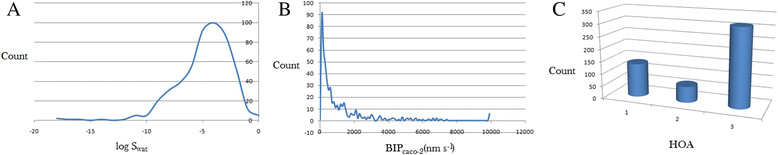
**Distribution of parameters for oral bioavailability and human oral absorption. (A)** log *S*_wat_, **(B)** BIP_Caco-2_ and **(C)** HOA. For the HOA parameter (C), 1, low absorption; 2, medium absorption; 3, high absorption.

In the assessment of oral bioavailability, 279 compounds showed no violation of the Rule of Three, while 422 compounds violated this rule only once, indicating that >80% of the compounds are predicted to be orally bioavailable. Regarding the individual parameters, the solubility parameter showed a significant performance, with about 80% of the compounds complying with the recommended range for 95% of known drugs (−6 ≤ log *S*_wat_ ≤ 0.5). In accordance to Jorgensen's ro3, 77.3% of the compounds had log *S*_wat_ > 5.7, while 91% complied with the BIP_Caco − 2_ > 22 nm s^−1^ criterion. The percentage of compounds complying with each of the other DMPK parameters has been shown in Table 2. The distribution curves of both parameters are shown in Figure 4A,B, along with the histogram for the human oral absorption (HOA) parameter. It was estimated that 312 compounds could have high HOA (attributed to a value of 3 on Figure 4C), while 64 compounds were estimated to have medium HOA (attributed to a value of 2) and 135 compounds were estimated to have low absorption. Estimations of the percentage of human oral absorption (PHOA) showed that 38.1% of the compounds could be absorbed at 100%, while 52.2% could be absorbed at >90% and 69.9% were estimated to have high PHOA (i.e., absorbed at >80%).

**Table 2:** **Mean values and** p**ercentage compliances of selected ADMET-related descriptors for 511 anti-malarial compounds from African medicinal plants**

Property^a^	Mean value	Percentage compliance
log *B/B*	−1.0	93.3
BIP_Caco-2_ (nm s^−1^)	1,139.0	14.1
*S*_mol_ (Å^2^)	641.5	95.1
*S*_mol, hfob_ (Å^2^)	350.0	95.5
*V*_mol_ (Å^3^)	1,202.3	94.5
log *S*_wat_ (*S* in mol L^−1^)	−4.3	79.3
log *K*_HSA_	0.3	83.6
MDCK	627.8	54.0
Ind_coh_	0.012	95.3
Glob	0.9	92.8
QP_polz_	39.6	94.1
logHERG	−4.6	57.3
log *K*_p_	−3.2	93.3
#metab	5.4	84.5

^a^The descriptors (entries in the first column and the recommended ranges for 95% of drugs) are defined in Additional file 3: Table S1.

### Assessment of the overall DMPK compliance by the drug-likeness parameter

**Figure 5:**
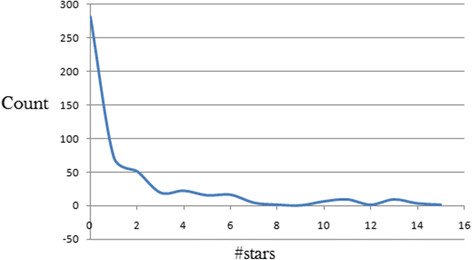
**Distribution curves for #stars for 511 anti-malarial compounds derived from African medicinal plants.**

The #stars parameter was also used to evaluate the overall drug-likeness of the anti-malarial compounds from African flora. The distribution curve for this parameter has been shown in Figure 5. The graph started with 0 violations of drug-likeness indicators (corresponding to a maximum number of 282 compounds) and went up to 15 violations. The steepness of the curve, however, showed that the compounds had an overall interesting drug-likeness. Additionally, 79.2% of the compounds violated only ≤2 out of the recommended range of values for the 24 essential descriptors of drug-likeness computed by QikProp in this study.

### Prediction of drug distribution and binding to human serum albumin and dermal penetration parameters

**Figure 6:**
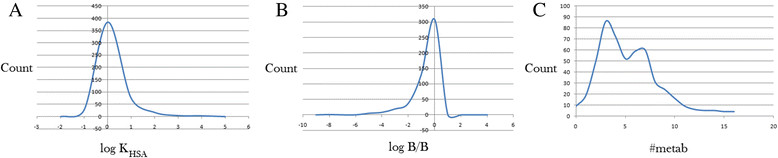
**Plot of the physicochemical descriptors used to predict DMPK. (A)** Predicted log *K*_HSA_ against count, **(B)** Predicted log *B*/*B* against count and **(C)** Predicted # metab against count.

The distribution of the compounds was simulated by calculation of binding affinities to human serum albumin (HSA). The distribution of log *K*_HSA_ gave a smooth Gaussian-shaped curve with a peak value at 0.5 log *K*_HSA_ units (Figure 6A), with 83.6% of the compounds falling within the required range for 95% of drugs. This is an indication that a significant proportion of the compounds are likely to circulate freely in the blood stream and hence reach the drug target sites.

The skin permeability parameter showed 93.3% compliance with 95% of drugs. The distribution curve was a Gaussian-shape centred on −2.5 log *K*_p_ units (Additional file 3: Figure S1), while the predicted maximum transdermal transport rates, *J*_m_ (in μ cm^−2^ h^−1^), were estimated to vary from 0 to about 1,640 units, with only five compounds having predicted value of *J*_m_ > 100 μ cm^−2^ h^−1^.

### Prediction of blood/brain barrier penetration and activity in the central nervous system

Access to the central nervous system was simulated by the log *B*/*B* parameter, while activity in the central nervous system was estimated by the CNS parameter (on the −2 = inactive and +2 = active scale). The log *B*/*B* plot (Figure 6B) showed a sharp peak at 0.5 log *B*/*B* units, with 93.3% compliance with 95% of drugs. On the other hand, only 3.5% of the compounds were estimated to have an activity in the central nervous system.

### Prediction of number of likely metabolic reactions

The number of likely metabolic steps was also computed by QikProp and plotted against the counts (Figure 6C). This parameter is often used to assess the likelihood of a molecule to easily gain access to the target site after entering the blood stream. The average estimated number of possible metabolic reactions was between 5 and 6, even though some of the compounds are likely to undergo as many as up to 16 metabolic reactions due to the complexity of some of the plant secondary metabolites. About 84% of the compounds are predicted to undergo the recommended number of metabolic steps (1 to 8 reactions),

### Prediction of toxicity by blockage of HERG K+ channel

**Figure 7:**
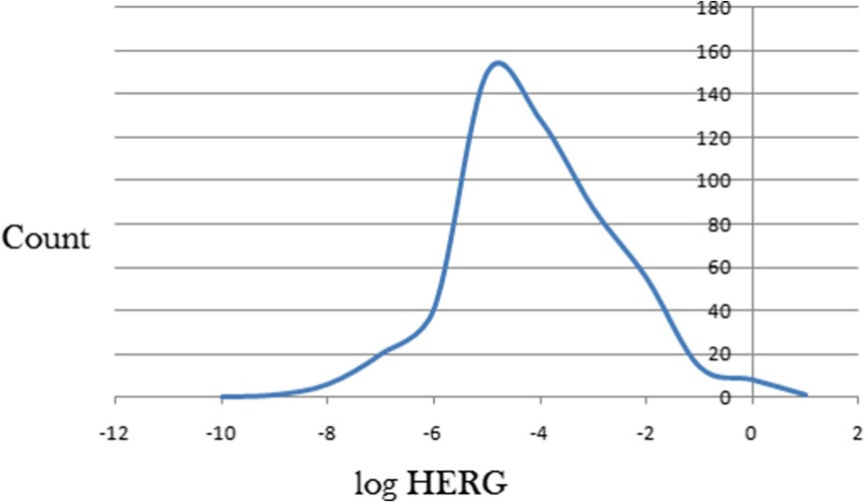
**A plot of predicted logHERG values for 511 anti-malarial compounds derived from the African flora.**

In this study, the estimated or predicted IC_50_ values for blockage of the HERG K^+^ channel have been used to model the drug toxicity *in silico*. The recommended range for predicted log IC_50_ values for blockage of HERG K^+^ channels (logHERG) is > −5. A distribution curve for the variation of the predicted logHERG is shown in Figure 7, which is a left-slanted Gaussian-shaped curve, with a peak at −5.5 logHERG units. It was observed that in general, this parameter is predicted to fall within the recommended range for about 57% of the compounds.

## Conclusions

In this study, the calculated physicochemical properties and indicators of drug-likeness have been used in the assessment of the ADMET/DMPK profiles of >500 compounds isolated from medicinal plants in Africa, which have exhibited from weak to high *in vitro* and/or *in vivo* anti-plasmodial/anti-malarial activity. The overall estimate gave good compliance with the computed parameters for 95% of known drugs, as well as with Lipinski' drug-likeness criteria. The results give good reason for further investigation of the suitability of anti-malarial compounds derived from African flora to be directly employed as drugs or as lead compounds from which new anti-malarial drugs could be developed. The generated 3D structures of the compounds have been included as a supplementary file (Additional file 2), while enquiries for the availability of compounds for screening purposes could be addressed to the p-ANAPL consortium [52], which has the mandate to collect compound samples from African flora for biological screening purposes.

## Additional files

## Electronic supplementary material

Additional file 1: List of journals consulted in the literature search. (DOCX 15 KB)

Additional file 2: **The 3D structures of the anti-malarial compounds isolated from African medicinal plants in this survey.** This file is saved in .sdf format (which can be viewed using a wide range of software, including MOE, LigandScout, Discovery Studio, and Maestro). (SDF 1 MB)

Additional file 3: Table S1.: Selected computed ADMET-related descriptors and their recommended ranges for 95% of known drugs. **Figure S1.** Distribution curves for the predicted skin permeability parameter. (DOCX 36 KB)

Below are the links to the authors’ original submitted files for images.

Authors’ original file for figure 1

Authors’ original file for figure 2

Authors’ original file for figure 3

Authors’ original file for figure 4

Authors’ original file for figure 5

Authors’ original file for figure 6

Authors’ original file for figure 7

## Abbreviations

3D: three-dimensional
ADMET: absorption, distribution, metabolism, excretion and toxicity
DMPK: drug metabolism and pharmacokinetics
HBA: hydrogen bond acceptors
HBD: hydrogen bond donors
log P: logarithm of the octan-1-ol/water partition coefficient
MDCK: Madin-Darby canine kidney
MW: molecular weight
NP: natural product
NRB: number of rotatable bonds
