# Supplementary material for: The potential of anti-malarial compounds derived from African medicinal plants, part III: an in silico evaluation of drug metabolism and pharmacokinetics profiling
Source: Org Med Chem Lett. 2014 Sep 5;4:6. doi: 10.1186/s13588-014-0006-x (PMC4970435; doi:10.1186/s13588-014-0006-x)
Supplement: Supplementary file 1 — Additional file 1: List of journals consulted in the literature search. (DOCX 15 KB) [file 13588_2014_6_MOESM1_ESM.docx]

# SUPPLEMENTARY MATERIAL

# The potential of anti-malarial compounds derived from African medicinal plants, part III: An *in silico* evaluation of drug metabolism and pharmacokinetics profiling

### Pascal Amoa Onguéné^1†^, Fidele Ntie-Kang^2,3†^, Lydia Likowo Lifongo^2^, Jean Claude Ndom^1^, Wolfgang Sippl^3^, Luc Meva’a Mbaze^1*^

^1^Department of Chemistry, Faculty of Science, University of Douala, P. O. Box 24157, Douala, Cameroon

^2^Chemical and Bioactivity Information Centre, Department of Chemistry, Faculty of Science, University of Buea, P. O. Box 63, Buea, Cameroon

^3^Department of Pharmaceutical Sciences, Martin-Luther University of Halle-Wittenberg, Wolfgang-Langenbeck Str. 4, 06120, Halle (Saale), Germany

^†^Equal contributors

^*^Corresponding author

Email addresses:

PAO: [amoapascal@yahoo.fr](mailto:amoapascal@yahoo.fr)

FNK: [ntiekfidele@gmail.com](mailto:ntiekfidele@gmail.com)

LLL: [llifongo@yahoo.com](mailto:llifongo@yahoo.com)

JCN: [ndomjefr@yahoo.fr](mailto:ndomjefr@yahoo.fr)

WS: [wolfgang.sippl@pharmazie.uni-halle.de](mailto:wolfgang.sippl@pharmazie.uni-halle.de)

LMM: [lmbazze@yahoo.fr](mailto:lmbazze@yahoo.fr)

List of Journals Consulted in in the survey of anti-malarial potential of African medicinal plants

| Journal type | List |
| --- | --- |
| International | *Acta Chimica Slovenica, Acta Crystallographica*, *African Journal of Biotechnology*, *African Journal of Health Sciences*, *African Health Sciences*, *Analytical Sciences*, *Annals of Tropical Medicine and Parasitology*, *Annals of Clinical Microbiology and Antimicrobials*, *Arkivoc*, *Asian Journal of Chemistry*, *Asian Journal of Traditional Medicine*, *Biochemical Systematics and Ecology*, *Bioorganic and Medicinal Chemistry*, *Bioorganic and Medicinal Chemistry Letters*, *Bioscience Biotechnology and Biochemistry*, *BMC Complementary and Alternative Medicine*, *BMC Research Notes*, *Boletín Latinoamericano y del Caribe de Plantas Medicinales y Aromáticas*, *Brazalian Journal Medical Biology Research*, *Bulletin of the Chemical Society of Ethiopia*, *Canadian Journal of Chemistry*, *Carbohydrate Research*, *Cell Division*, *Chemistry and Biodiversity*, *Chemical and Pharmaceutical Bulletin*, *Chemistry of Natural Compounds*, *European Journal of Pharmacology, European Journal of Plant Pathology*, *Fitoterapia*, *Greener Journal of Biological Sciences, Helvetica Chimica Acta*, *Indian Journal of Pharmacology*, *Inflammopharmacology*, *International Journal of Pharmacy and Pharmaceutical Sciences*, *International Journal Antimicrobial Agents*, *International Journal of Mass Spectrometry*, *Iranian Journal of Medical Sciences*, *Journal of Antibiotics*, *Journal of Brazilian Chemical Society*, *Journal of Medicinal Chemistry*, *Journal of Natural Products*, *Journal of Organic Chemistry*, *Journal of Asian Natural Products Research*, *Journal of Ethnopharmacology*, *Journal of the American Oil Chemistry Society*, *Leukemia Research*, *Malaria Journal*, *Molecules*, *Natural Product Communications*, *Natural Product Letters*, *Natural Product Research*, *Natural Product Science*, *Pakistani Journal of Medical Science*, *Parasitology Research*, *Pharmacologia*, *Pharmacologyonline*, *Pharmacopée de Médecine Traditionnelle Africaine*, *Pharmazie*, *Phytochemistry*, *Phytochemistry Letters*, *Phytochemical Analysis*, *Pharmaceutical Biology*, *Phytotherapy Research*, *Phytomedicine*, *Planta Medica*, *PLoS One*, *Pure and Applied Chemistry*, *Rasayan Journal of Chemistry*, *Records of Natural Products*, *Research Journal in Phytochemistry*, *South African Journal of Botany*, *Talanta*, *Tetrahedron*, *Tetrahedron Letters*  and *Zeitschrift für Naturforschung*. |
| Cameroonian | *Cameroonian Journal of Experimental Biology,* *Journal of the Cameroonian Academy of Sciences*, *Cameroon Journal of Biosciences* and *Les Annales des la Faculté des Sciences des l’Université de Yaoundé I.* |
