# Supplementary material for: The potential of anti-malarial compounds derived from African medicinal plants, part III: an in silico evaluation of drug metabolism and pharmacokinetics profiling
Source: Org Med Chem Lett. 2014 Sep 5;4:6. doi: 10.1186/s13588-014-0006-x (PMC4970435; doi:10.1186/s13588-014-0006-x)
Supplement: Supplementary file 3 — Additional file 3: Table S1.: Selected computed ADMET-related descriptors and their recommended ranges for 95% of known drugs. Figure S1. Distribution curves for the predicted skin permeability parameter. (DOCX 36 KB) [file 13588_2014_6_MOESM3_ESM.docx]

SUPPLEMENTARY DATA

# The potential of anti-malarial compounds derived from African medicinal plants, part III: An *in silico* evaluation of drug metabolism and pharmacokinetics profiling

### Pascal Amoa Onguéné^1†^, Fidele Ntie-Kang^2,3†^, Lydia Likowo Lifongo^2^, Jean Claude Ndom^1^, Wolfgang Sippl^3^, Luc Meva’a Mbaze^1*^

^1^Department of Chemistry, Faculty of Science, University of Douala, P. O. Box 24157, Douala, Cameroon

^2^Chemical and Bioactivity Information Centre, Department of Chemistry, Faculty of Science, University of Buea, P. O. Box 63, Buea, Cameroon

^3^Department of Pharmaceutical Sciences, Martin-Luther University of Halle-Wittenberg, Wolfgang-Langenbeck Str. 4, 06120, Halle (Saale), Germany

^†^Equal contributors

^*^Corresponding author

Email addresses:

PAO: [amoapascal@yahoo.fr](mailto:amoapascal@yahoo.fr)

FNK: [ntiekfidele@gmail.com](mailto:ntiekfidele@gmail.com)

LLL: [llifongo@yahoo.com](mailto:llifongo@yahoo.com)

JCN: [ndomjefr@yahoo.fr](mailto:ndomjefr@yahoo.fr)

WS: [wolfgang.sippl@pharmazie.uni-halle.de](mailto:wolfgang.sippl@pharmazie.uni-halle.de)

LMM: [lmbazze@yahoo.fr](mailto:lmbazze@yahoo.fr)

**Table S1:** **Selected computed ADMET-related descriptors and their recommended ranges for 95% of known drugs**

| **Property** | **Description** | **DMPK significance** | **Recommended range** |
| --- | --- | --- | --- |
| Ro5 | Number of violations of Lipinski’s “Rule of Five” [1] | Oral bioavailability (depends on MW, log P, HBD and HBA). Compounds which comply to this rule are considered to be drug-like | ≤ 4 |
| Ro3 | Number of violations of Jorgensen’s “Rule of Three” [2] | Oral bioavailability (depends on log S*_wat_*, *BIP_caco_*_–2_ and number of primary metabolites). Compounds with fewer (and preferably no) violations of these rules are more likely to be orally bioavailable. | ≤ 3 |
| MW | Molecular weight |  | 130 to 725 Da |
| log P | Logarithm of octan-1-ol/water partition coefficient |  | -2 to 6.5 |
| HBA | Number of hydrogen bond acceptors |  | 2 to 20 |
| HBD | Number of hydrogen bond donors |  | 0 to 6 |
| NRB | Number of rotatable single bonds |  | 0 to 15 |
| ^*^#stars | “drug-likeness” parameter [2] | Comparison to 95% of known drugs | 0 to 5 |
| HOA | Predicted human oral absorption parameter | Oral absorption | 1 low, 2 medium, 3 high |
| PHOA | Predicted percentage human oral absorption parameter | Oral absorption | 80% high, < 25% poor |
| S*_mol_* | the total solvent-accessible molecular surface, in Å^2^ (probe radius 1.4 Å) |  | 300 to 1000 Å^2^ |
|  |  |  |  |
| S*_mol,hfob_* | the hydrophobic portion of the solvent-accessible molecular surface, in Å^2^ (probe radius 1.4 Å) |  | 0 to 750 Å^2^ |
| V*_mol_* | the total volume of molecule enclosed by solvent-accessible molecular surface, in Å^3^ (probe radius 1.4 Å) |  | 500 to 2000 Å^3^ |
| log S*_wat_* | the logarithm of aqueous solubility [3-4] | Drug absorption | −6.0 to 0.5 |
| log *K_HSA_* | the logarithm of predicted binding constant to human serum albumin [5] | Drug distribution | −1.5 to 1.2 |
| log *B/B* | the logarithm of predicted blood/brain barrier partition coefficient [6-8] | Access of drug to central nervous system (CNS) | −3.0 to 1.0 |
| *BIP_caco_*_–2_ | the predicted apparent Caco-2 cell membrane permeability, in nm s^-1^ (in Boehringer–Ingelheim scale, [9-11]) | permeability of the gut-blood barrier | < 5 low, > 100 high |
| *MDCK* | the predicted apparent Madin-Darby canine kidney cell permeability in nm s^-1^ [10] |  | < 25 poor, > 500 great |
| Ind_coh_ | the index of cohesion interaction in solids, calculated from the number of hydrogen bond acceptors (HBA), donors (HBD) and the surface area accessible to the solvent, SASA (*S_mol_*) by the relation $\mathrm{Ind}_{\mathrm{coh}}=HBA\times\sqrt{\mathrm{HBD}}/S_{\mathrm{mol}}$ [3] | influences drug solubility | 0.0 to 0.05 |
| Glob | the globularity descriptor, Glob = (4*πr*^2^)/*S_mol_*, where *r* is the radius of the sphere whose volume is equal to the molecular volume |  | 0.75 to 0.95 |
| *QP_polrz_* | the predicted polarizability |  | 13.0 to 70.0 |
| log *HERG* | the predicted IC_50_ value for blockage of HERG K^+^ channels [12-13] | Drug toxicity | concern < −5 |
| log *K*_p_ | the predicted skin permeability [14-15] | Drug distribution | −8.0 to −1.0 |
| *#metab* | the number of likely metabolic reactions | Drug metabolism | 1 to 8 |

*The following properties and descriptors are included in the determination of #stars: MW, dipole moment, the PM3 calculated ionization potential, the PM3 calculated electron affinity, S*_mol_*, S*_mol,hfob_*, the Hydrophilic component of the solvent accessible surface area (solvent accessible surface area on N, O, and H on heteroatoms), the π (carbon and attached hydrogen) component of the solvent accessible surface area, the Weakly polar component of the solvent accessible surface area (halogens, P, and S), the Van der Waals surface area of polar nitrogen and oxygen atoms. V*_mol_*, NRB, HBD, HBA, glob, QP*_polrz_*, the predicted hexadecane/gas partition coefficient, the Predicted octanol/gas partition coefficient, Predicted water/gas partition coefficient, log P, log S*_wat_*, log *K_HSA_*, *BIP_caco_*_–2_, and #metab


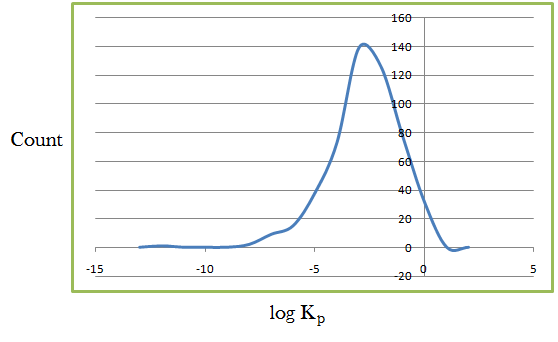


## Figure S1 - Distribution curves for the predicted skin permeability parameter.

**References**

[1] Lipinski CA, Lombardo F, Dominy BW, Feeney PJ: **Experimental and computational approaches to estimate solubility and permeability in drug discovery and development settings.** *Adv Drug Delivery Rev* 1997, **23**:3-25

[2] Schrödinger Press: QikProp 3.4 User Manual, LLC, New York, NY, 2011.

[3] Jorgensen WL, Duffy EM: **Prediction of drug solubility from Monte Carlo simulations.** *Bioorg Med Chem Lett* 2000, **10**:1155-1158

[4] Jorgensen WL, Duffy EM: **Prediction of drug solubility from structure.** *Adv Drug Deliv Rev* 2002, **54**:355-366

[5] Colmenarejo G, Alvarez-Pedraglio A, Lavandera JL: **Cheminformatic models to predict binding affinities to human serum albumin.** *J Med Chem* 2001, **44**:4370-4378

[6] Luco JM: **Prediction of brain-blood distribution of a large set of drugs from structurally derived descriptors using partial least squares (PLS) modelling.** *J Chem Inf Comput Sci* 1999, **39**:396-404

[7] Ajay, Bermis GW, Murkco MA: **Designing libraries with CNS activity.** *J Med Chem* 1999, **42**:4942-4951

[8] Kelder J, Grootenhuis PD, Bayada DM, Delbresine LP, Ploemen JP: **Polar molecular surface as a dominating determinant for oral absorption and brain pernetration of drugs.** *Pharm Res* 1999, **16**:1514-1519

[9] Yazdanian M, Glynn SL, Wright JL, Hawi A: **Correlating partitioning and caco-2 cell permeability of structurally diverse small molecular weight compounds.** *Pharm Res* 1998, **15**:1490-1494

[10] Irvine JD, Takahashi L, Lockhart K, Cheong J, Tolan JW, Selick HE, Grove JR: **MDCK (Madin-Darby canine kidney) cells: a tool for membrane permeability screening.** *J Pharm Sci* 1999, **88**:28-33

[11] Stenberg P, Norinder U, Luthman K, Artursson P: **Experimental and computational screening models for the prediction of intestinal drug absorption.** *J Med Chem* 2001, **44**:1927-1937

[12] Cavalli A, Poluzzi E, De Ponti F, Recanatini M: **Toward a pharmacophore for drugs inducing the long QT syndrome: Insights from a CoMFA Study of HERG K^+^ channel blockers.** *J Med Chem* 2002, **45**:3844-3853

[13] De Ponti F, Poluzzi E, Montanaro N: **Organising evidence on QT prolongation and occurrence of Torsades de Pointes with non-antiarrhythmic drugs: a call for consensus.** *Eur J Clin Pharmacol* 2001, **57**:185-209

[14] Potts RO, Guy RH: **Predicting skin permeability.** *Pharm Res* 1992, **9**:663-669

[15] Potts RO, Guy RH: **A predictive algorithm for skin permeability: The effects of molecular size and hydrogen bond activity.** *Pharm Res* 1995, **12**:1628-1633
