# Supplementary figures and images for: The potential of anti-malarial compounds derived from African medicinal plants, part III: an in silico evaluation of drug metabolism and pharmacokinetics profiling
Source: Org Med Chem Lett. 2014 Sep 5;4:6. doi: 10.1186/s13588-014-0006-x (PMC4970435; doi:10.1186/s13588-014-0006-x)

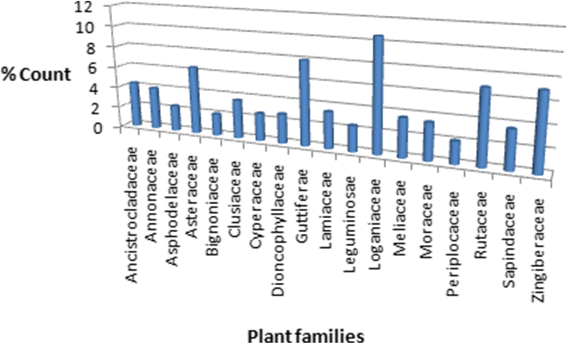

Supplement: Supplementary file 4 — Authors’ original file for figure 1 [file 13588_2014_6_MOESM4_ESM.gif]

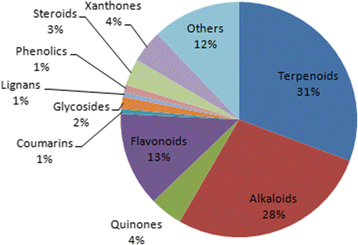

Supplement: Supplementary file 5 — Authors’ original file for figure 2 [file 13588_2014_6_MOESM5_ESM.gif]

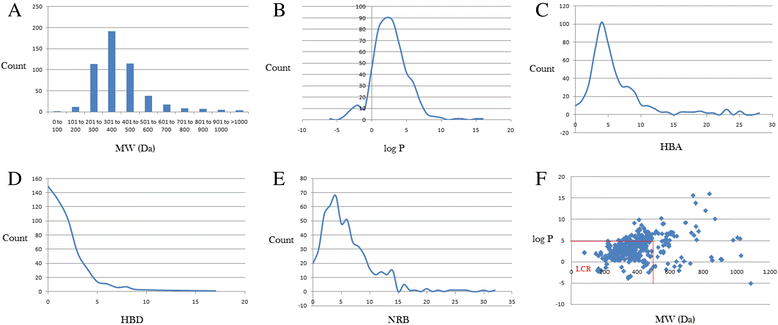

Supplement: Supplementary file 6 — Authors’ original file for figure 3 [file 13588_2014_6_MOESM6_ESM.gif]

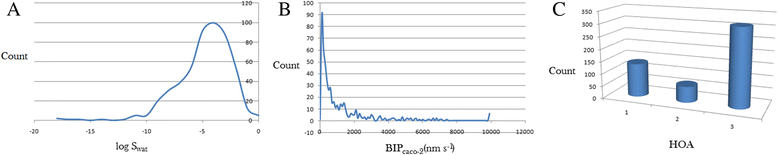

Supplement: Supplementary file 7 — Authors’ original file for figure 4 [file 13588_2014_6_MOESM7_ESM.gif]

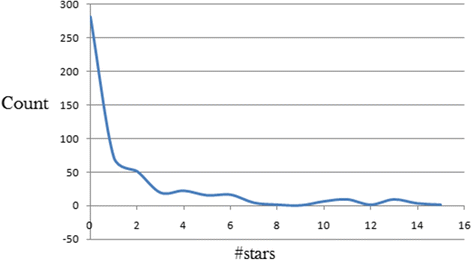

Supplement: Supplementary file 8 — Authors’ original file for figure 5 [file 13588_2014_6_MOESM8_ESM.gif]

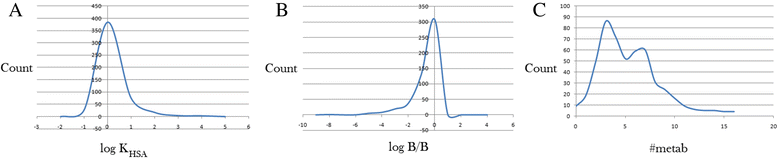

Supplement: Supplementary file 9 — Authors’ original file for figure 6 [file 13588_2014_6_MOESM9_ESM.gif]

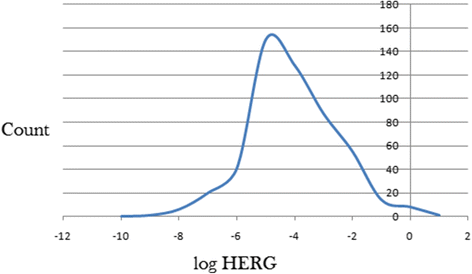

Supplement: Supplementary file 10 — Authors’ original file for figure 7 [file 13588_2014_6_MOESM10_ESM.gif]
